# Mechanism-Driven Strength–Conductivity Synergy in Hypereutectic Al-Si Alloys Reinforced with Interface-Engineered Ni-Coated CNTs

**DOI:** 10.3390/ma18153647

**Published:** 2025-08-03

**Authors:** Xuexuan Yang, Yulong Ren, Peng Tang, Jun Tan

**Affiliations:** 1 Guangxi Key Laboratory of Processing for Non-Ferrous Metals and Featured Materials, School of Resources, Environment and Materials, Guangxi University, Nanning 530004, China; 2039200217@st.gxu.edu.cn (X.Y.); 2415301056@st.gxu.edu.cn (Y.R.); 2 Singapore Centre for 3D Printing, School of Mechanical and Aerospace Engineering, Nanyang Technological University, Singapore 639798, Singapore; 3 College of Materials Science and Engineering, Chongqing University, Chongqing 400044, China

**Keywords:** Ni-CNTs, Al-Si alloys, strengthening mechanisms, electrical conductivity, intermetallic phases

## Abstract

Secondary hypereutectic Al-Si alloys are attractive for sustainable manufacturing, yet their application is often limited by low strength and electrical conductivity due to impurity-induced microstructural defects. Achieving a balance between mechanical and conductive performance remains a significant challenge. In this work, nickel-coated carbon nanotubes (Ni-CNTs) were introduced into secondary Al-20Si alloys to tailor the microstructure and enhance properties through interfacial engineering. Composites containing 0 to 0.4 wt.% Ni-CNTs were fabricated by conventional casting and systematically characterized. The addition of 0.1 wt.% Ni-CNTs resulted in the best combination of properties, with a tensile strength of 170.13 MPa and electrical conductivity of 27.60% IACS. These improvements stem from refined α-Al dendrites, uniform eutectic Si distribution, and strong interfacial bonding. Strengthening was achieved through grain refinement, Orowan looping, dislocation generation from thermal mismatch, and the formation of reinforcing interfacial phases such as AlNi_3_C_0_._9_ and Al_4_SiC_4_. At higher Ni-CNT contents, property degradation occurred due to agglomeration and phase coarsening. This study presents an effective and scalable strategy for achieving strength–conductivity synergy in secondary aluminum alloys via nanoscale interfacial design, offering guidance for the development of multifunctional lightweight materials.

## 1. Introduction

Secondary aluminum production has gained an increasing amount of attention in recent years due to its substantial energy savings and environmental benefits. Compared to primary aluminum, secondary aluminum requires only about 5% of the energy and significantly reduces carbon emissions [1,2]. Among its various applications, hypereutectic Al-Si alloys, particularly those containing approximately 20 wt.% silicon, are widely used in critical automotive and aerospace components due to their excellent wear resistance [3,4], low thermal expansion [5,6], and good castability [7]. However, the inherent variability in the composition of secondary aluminum feedstocks, especially with respect to impurity elements such as iron (Fe), often leads to microstructural inhomogeneity and inconsistent mechanical performance [8,9]. These challenges restrict the broader application of secondary Al-Si alloys in reliability-critical structural environments. Therefore, developing effective reinforcement strategies to stabilize the microstructure and enhance both mechanical and functional properties is of considerable practical and scientific interest.

Carbon nanotubes (CNTs), due to their exceptional tensile strength, elastic modulus, high aspect ratio, and excellent thermal and electrical conductivities, have been proposed as high-performance reinforcements for aluminum matrix composites [10]. Nonetheless, their limited wettability with the Al matrix and strong tendency to agglomerate significantly impede their dispersion and interfacial load transfer efficiency [11]. To overcome these issues, surface modifications such as metal coatings (e.g., Ni [12,13], CuO [14], Cu [15]), ceramic interlayers (e.g., SiC [16], TiC [17]), and graphene oxide [18] have been explored to enhance interfacial bonding and inhibit detrimental interfacial reactions. Among these approaches, nickel-coated CNTs (Ni-CNTs) have emerged as a particularly promising solution. The Ni coating enhances interfacial wettability and bonding between CNTs and the aluminum matrix, while simultaneously suppressing the formation of brittle Al_4_C_3_ phases. Furthermore, Ni facilitates the in situ formation of strengthening intermetallic compounds such as Al_3_Ni [19] and NiSi_2_ [20], contributing to improved thermal stability and mechanical reinforcement.

Recent studies have highlighted the effectiveness of Ni-CNTs in improving the performance of Al-based composites. For instance, Zhang et al. [21] reported significant enhancements in yield strength, tensile strength, ductility, and microhardness. Ding et al. [22] demonstrated favorable mechanical and thermal properties in Al-Si composites reinforced with 2 wt.% CNTs. Yuan et al. [23], using laser cladding techniques, showed that 1 wt.% Ni-CNTs could enhance wear and corrosion resistance. Lv et al. [24] found that Ni-CNTs with a content of 0.5 wt% significantly improved the hardness, compressive strength, and compressive plasticity of a high-entropy alloy. Moreover, Kadhim [25] and Zhang [11] systematically analyzed the synergistic strengthening mechanisms of Ni-CNTs, including stress transfer, dislocation generation, and fracture behavior regulation. Fan et al. [26] explained the nucleation mechanisms of aluminum alloy particles and the second phase through the first-phase principles. Shi et al. [27] pointed out the crucial role of the cellular structure surrounded by the interconnected silicon-rich eutectic network in the Al-Si alloy and the second phase in the tensile behavior. Despite these advances, most existing research focuses on primary aluminum or idealized laboratory conditions. In contrast, secondary hypereutectic Al-20Si alloys inherently contain residual impurity elements such as Fe and N. The effects of Ni-CNTs on microstructural regulation and the associated strengthening mechanisms remain insufficiently understood.

In this study, a secondary Al-20Si alloy was selected as the matrix to systematically investigate the effects of Ni-CNT additions (0.1–0.4 wt.%) on microstructure evolution and composite performance. Particular emphasis was placed on strengthening mechanisms such as grain refinement, load transfer, and thermal mismatch-induced dislocation generation. In addition, the potential synergistic interaction between Ni-CNTs and native Ni/Fe components in the matrix was explored. This work aims to establish an optimal reinforcement range and to provide insights into the design of advanced aluminum matrix composites with integrated structural and functional performance.

## 2. Materials and Methods

In this study, a secondary hypereutectic Al-20Si alloy was used as the matrix material. Intermediate alloys, including Al-10Fe, Al-10Ni, and Al-3Cu, along with pure Zn, were employed to adjust the nominal composition and the actual composition, as listed in Table 1. The actual composition in the table was obtained by testing with the Thermo Scientific ARL iSpark 8860 (Thermo Fisher Scientific, Waltham, MA, USA). Due to the scarcity of inner electrons in carbon atoms, they are difficult to excite by conventional X-ray excitation sources. Therefore, the actual composition of nickel-plated CNTs will be ignored in the table.

**Figure 1:**
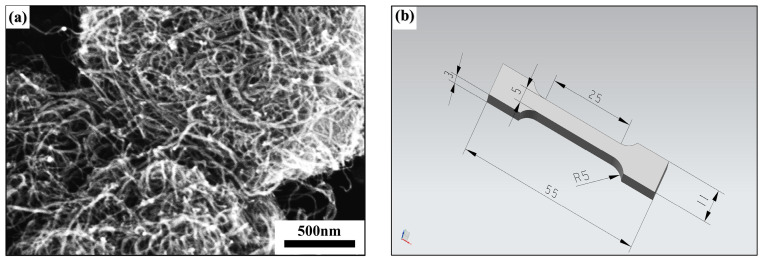
(**a**) SEM image of nickel-coated carbon nanotubes (Ni-CNTs); (**b**) schematic diagram of the tensile specimen geometry (mm).

Nickel-coated carbon nanotubes (Ni-CNTs) were utilized as the reinforcing phase. These Ni-CNTs were fabricated via chemical electroless plating, in which a uniform nickel layer was deposited on the CNT surface to enhance wettability and interfacial compatibility with the aluminum melt. Compared to untreated CNTs, Ni-CNTs exhibit superior dispersion and metallurgical bonding within the Al matrix. The morphology of Ni-CNTs was observed using a Hitachi high-resolution field emission scanning electron microscope SU8020 (Hitachi High Technologies, Tokyo, Japan) at 60,000 magnification and operating at 10 kV, as shown in Figure 1a. The average diameter of the nanotubes is approximately 20 ± 10 nm, with lengths of ~20 ± 10 μm and a purity of 98%.

**Figure 2:**
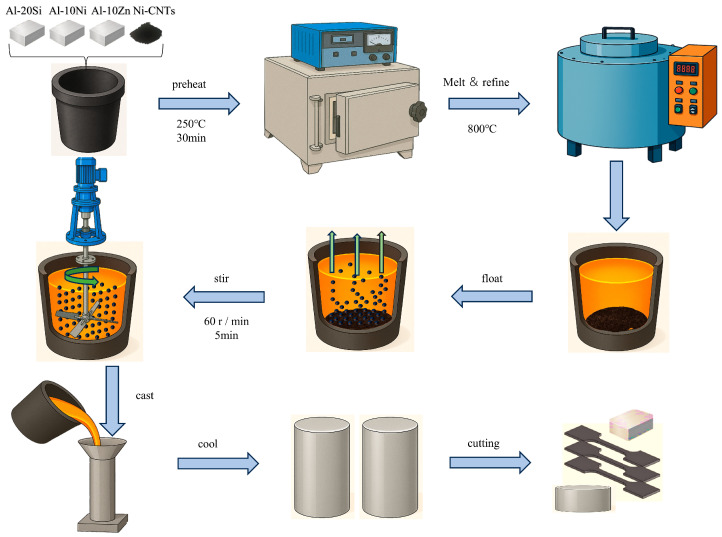
Flowchart of the experimental procedure for alloy preparation and characterization.

The fabrication process is illustrated in Figure 2. Melting and casting were conducted using an SG2-7.5-10 resistance furnace(Shanghai AS Electric Furnace Factory, Shanghai, China). Graphite crucibles were used for melting, and cast-iron molds were used for shaping the specimens. Prior to melting, the crucibles and molds were preheated to 523 K to minimize thermal gradients. The secondary Al-20Si base alloys and alloying additives (Ni-CNTs, Al-Ni master alloys, and their combined additions) were sequentially loaded into the crucible, with the Ni-CNT powder being placed at the bottom to facilitate melting infiltration. The charge was then heated to 750 °C and held until fully molten. Mechanical stirring was performed at 60 rpm for 5 min to promote dispersion of the Ni-CNTs within the melt. Subsequently, a fluxing agent was added to the melt surface, and the mixture was held static for 25 min to allow for homogenization. A refining agent was added just before slag removal to enhance melt cleanliness. Once the temperature decreased to 720 °C, slag was removed, and the melt was gravity-cast into preheated molds. The castings were then allowed to solidify under ambient conditions. Five different Ni-CNT content levels were investigated, 0, 0.1, 0.2, 0.3, and 0.4 wt.%, and designated as samples #1 through #5, respectively.

Cylindrical castings (20 mm in diameter and 130 mm in length) were sectioned 20 mm from the bottom for microstructural and property analyses. Standard metallographic procedures were followed, including sequential grinding, polishing, and etching with Keller’s reagent (for 5–8 s) to reveal the microstructure. Microscopic structure observation was conducted using the Zeiss Axio Observer optical microscope (Carl Zeiss AG, Oberkochen, Germany). And using the Hitachi high-resolution field emission SEM SU8020 and its accompanying energy spectrum, the microscopic structure surfaces were scanned and analyzed point by point. The electrical conductivity was measured using a Sigma 2008A digital conductivity meter(Xiamen Tianyan Instrument Co., LTD, Xiamen, China). Each data point represents the average of ten repeated measurements to ensure accuracy. The preparation of the tensile specimens followed the ASTM E8/E8M standard [28] and was processed according to the geometric shape shown in Figure 1b. These specimens were tested using the Instron 8801 electronic universal testing machine. The testing machine operated at a constant strain rate of 0.5 mm per minute. After the test, the fracture surface was observed using SEM (Hitachi High Technologies, Tokyo, Japan) to analyze its morphology. Qualitative analysis of the experimental samples was carried out using a Bruker XRD Goniomerter (A24A10)(Bruker AXS GmbH, Karlsruhe, Germany). The hardness test was conducted using the HV-1000SPTA-type micro-Vickers hardness tester(Veiyee, Laizhou, China) with a load of 500 g. For each sample, 5 hardness values were measured, and the average value was taken as the hardness value of that sample.

## 3. Results

### 3.1. Microstructural Evolution Induced by Ni-CNT Addition

**Figure 3:**
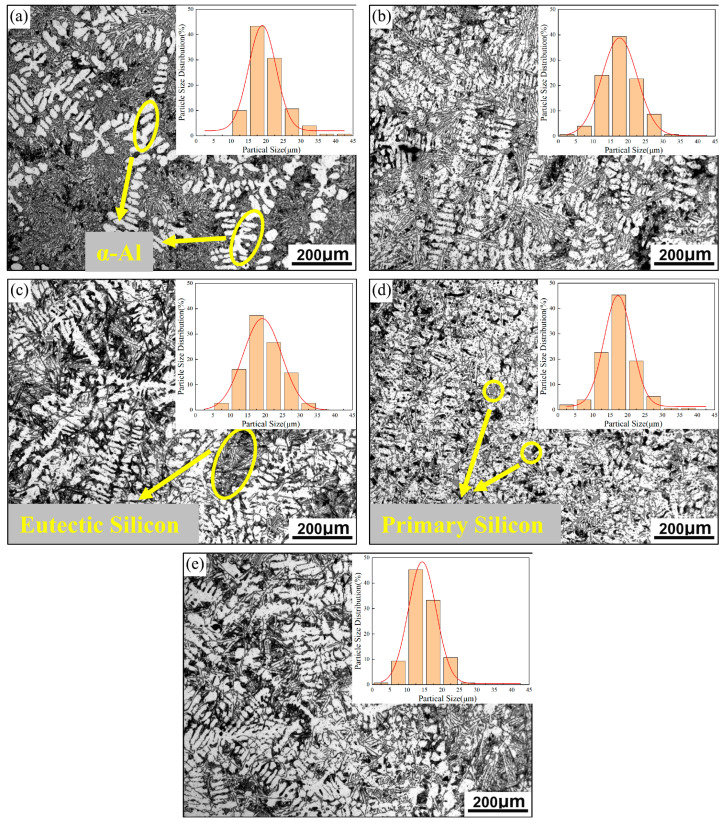
Optical micrographs of hypereutectic Al-Si alloys with varying Ni-CNT contents: (**a**) 0 wt.%, (**b**) 0.1 wt.%, (**c**) 0.2 wt.%, (**d**) 0.3 wt.%, and (**e**) 0.4 wt.%.

**Figure 4:**
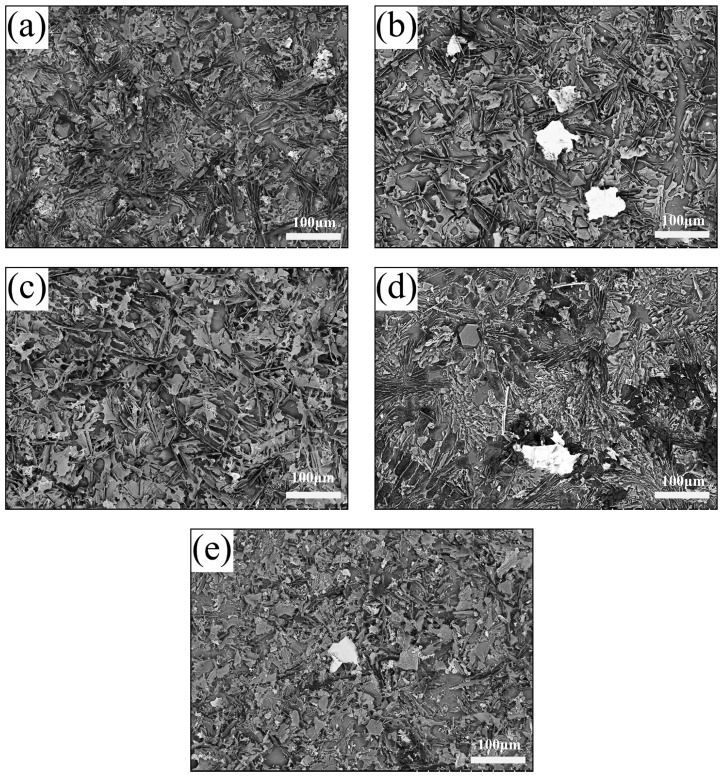
SEM images of etched hypereutectic Al-Si alloys with different Ni-CNT additions: (**a**) 0 wt.%, (**b**) 0.1 wt.%, (**c**) 0.2 wt.%, (**d**) 0.3 wt.%, and (**e**) 0.4 wt.%.

Figure 3 illustrates the optical microstructures of the hypereutectic Al-Si alloys containing varying amounts of Ni-CNTs. The base alloy’s composition lies near the eutectic point in the Al-Si binary phase diagram, and thus the microstructure comprises primary α-Al dendrites, primary Si particles, and eutectic mixtures of α-Al and Si. In the as-cast alloy without Ni-CNTs (Figure 3a), coarse α-Al dendrites and irregularly distributed eutectic Si phases dominate the structure. The eutectic Si appears as coarse, interconnected needle-like structures that are concentrated at α-Al grain boundaries, while the primary Si phase is observed as polyhedral or blocky white dendrites embedded in the matrix. With the incremental addition of Ni-CNTs, significant microstructural refinement is observed. At a Ni-CNT content of 0.1 wt.% (Figure 3b), the α-Al dendrites exhibit pronounced grain refinement, and eutectic Si transforms into a finer, skeleton-like morphology with a more homogeneous distribution throughout the matrix. This refinement can be attributed to the potent heterogeneous nucleation sites provided by the uniformly dispersed Ni-CNTs during solidification. As the addition increases further to 0.2 wt.% and 0.3 wt.% (Figure 3c,d), the α-Al dendrites continue to refine, while the eutectic Si becomes more fibrous and elongated. Notably, in the 0.4 wt.% Ni-CNTs (Figure 3e), the eutectic Si volume fraction reduces, and the amount and size of primary Si particles increase significantly, suggesting possible solute segregation and local undercooling variation during solidification. A statistical analysis of the secondary dendrite arm spacing (SDAS) of α-Al, derived from 150 measurements per sample and shown in the inset of Figure 3, reveals a decreasing trend in SDAS with increasing Ni-CNT addition. For the unreinforced sample (#1), most SDAS values fall within 15–25 µm, whereas in sample #5 (0.4 wt.% Ni-CNTs), over 75% of the α-Al dendrites exhibit SDAS values in the range of 10–20 µm. Simultaneously, the characteristic length of eutectic Si also decreases. These results confirm the grain-refining effect of Ni-CNTs, which is beneficial for enhancing mechanical performance. Further insights into microstructural evolution are provided by SEM analysis of etched samples (Figure 4). In the unreinforced alloy (Figure 4a), long, sheet-like eutectic Si with a dispersed, non-uniform distribution is evident. With the addition of 0.1 wt.% Ni-CNTs (Figure 4b), the volume fraction of coarse Si decreases, and fine eutectic Si precipitates are observed. The white phase corresponds to partially uncorroded α-Al matrix. In the 0.2 wt.% Ni-CNTs (Figure 4c), eutectic Si shows a tendency to coarsen, with a more prominent sheet-like morphology. For 0.3 wt.% and 0.4 wt.% additions (Figure 4d,e), the primary Si becomes more dominant, displaying larger sizes and increased agglomeration, while eutectic Si appears reduced in both size and quantity. These observations further validate the trend observed in the optical micrographs.

**Figure 5:**
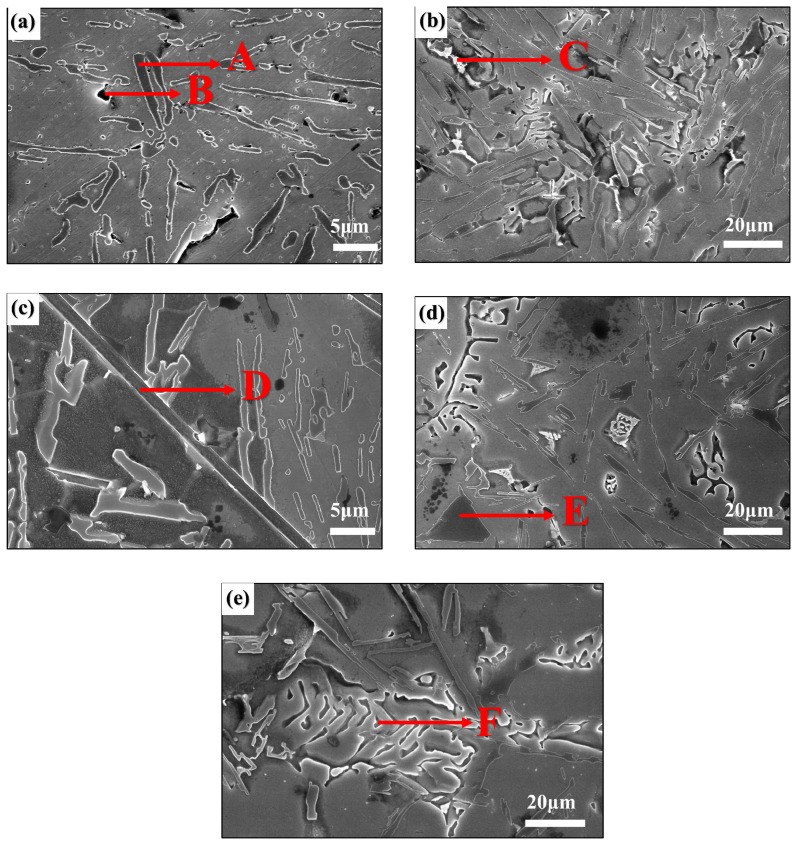
SEM images and corresponding EDS mappings of Al-Si alloys with various Ni-CNT contents: (**a**) 0 wt.%, (**b**) 0.1 wt.%, (**c**) 0.2 wt.%, (**d**) 0.3 wt.%, and (**e**) 0.4 wt.%.

To identify the key phases in the Ni-modified hypereutectic Al-Si alloys, detailed SEM and EDS analyses were conducted (Figure 5 and Table 2). Multiple intermetallic compounds were identified based on their morphology and elemental composition, consistent with previous research [19,25,29,30,31,32,33]. The eutectic Al-Si structure appears as fibrous or short rod-like features (Point A, Figure 5a). The black blocky phases observed at Points B and F (Figure 5e) are identified as α-Al_8_Fe_2_Si, which is generally considered a less detrimental Fe-rich phase. Point C in Figure 5b shows a phase enriched with Ni and Fe, likely corresponding to Al_9_FeNi and Al_3_Ni intermetallic phases, both of which serve as beneficial reinforcement phases in Al alloys. Point D in Figure 5c corresponds to a long rod-like β-Al_5_FeSi phase, known for its brittleness and detrimental impact on ductility. Point E in Figure 5d is identified as primary Si, characterized by its angular, blocky morphology. The coexistence of Fe-Ni intermetallic, α-Al_8_Fe_2_Si, and β-Al_5_FeSi phases indicates a complex reaction environment influenced by the Ni-CNTs during melt solidification. Trace elements such as Cu and Zn were also detected by EDS, possibly due to minor alloying reactions or impurities in raw materials.

### 3.2. Mechanical Properties and Fracture Morphology

**Figure 6:**
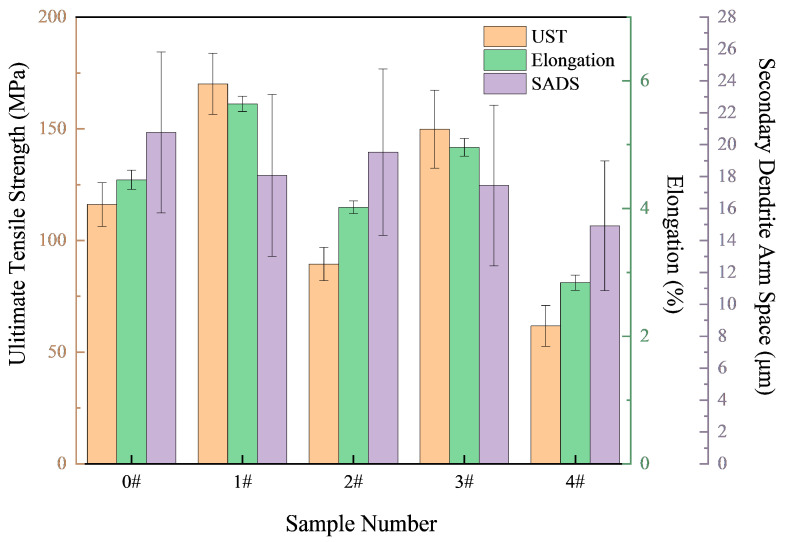
Effect of Ni-CNT content on secondary dendrite arm spacing (SDAS), ultimate tensile strength (UTS), and elongation (EL) of hypereutectic Al-Si alloys.

**Figure 7:**
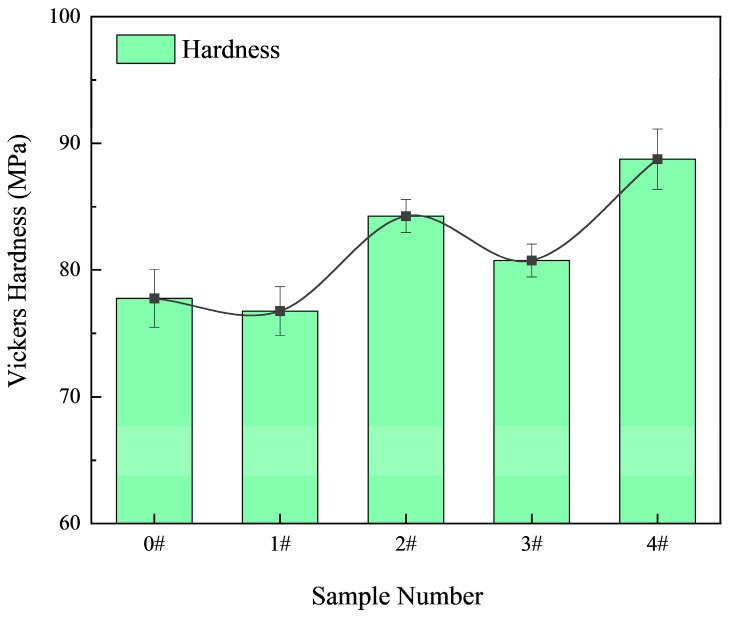
Variation in Vickers hardness of hypereutectic Al-Si alloys with different Ni-CNT additions.

**Figure 8:**
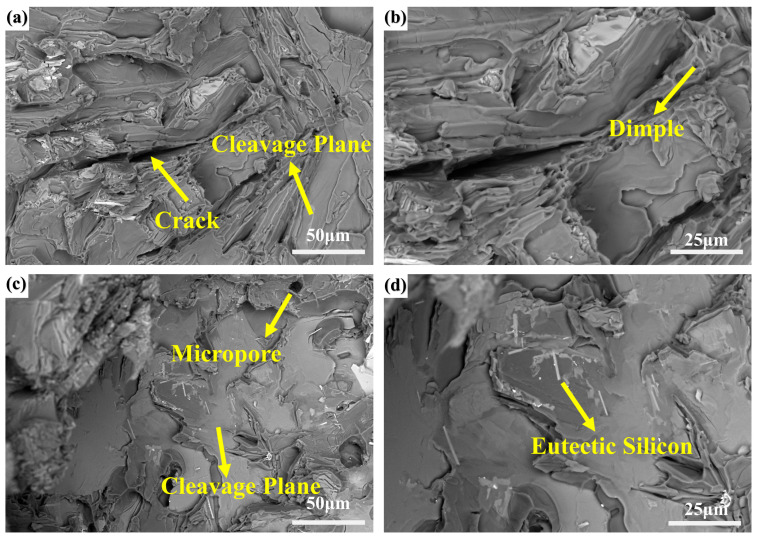
Fracture morphologies of hypereutectic Al-Si alloys: (**a**,**b**) 0.1 wt.% Ni-CNTs; (**c**,**d**) 0.2 wt.% Ni-CNTs.

The influence of Ni-CNT additions on the mechanical performance of hypereutectic Al-Si alloys is summarized in Figure 6, Figure 7 and Figure 8, which demonstrate that increasing the Ni-CNT content from 0 to 0.4 wt.% leads to a progressive decrease in the secondary dendrite arm spacing (SDAS), accompanied by a non-monotonic variation in tensile strength and elongation. Specifically, the alloy with 0.1 wt.% Ni-CNTs exhibits the highest tensile strength and elongation, reaching 170.13 MPa and 5.64%, respectively. These improvements are closely associated with the significant microstructural refinement and more homogeneous distribution of eutectic silicon observed in Figure 3b, where the eutectic network transitions to a fine and uniformly dispersed morphology, and the α-Al dendrites are substantially refined. This enhancement is attributed to the synergistic nucleation effects of both nickel and carbon components in Ni-CNTs, which serve as effective heterogeneous nucleation sites during solidification. Such nucleation promotes the formation of fine grains, reduces thermal gradients, and minimizes solute segregation. However, with further increases in Ni-CNT content (≥0.2 wt.%), a decline in both tensile strength and elongation is observed. This deterioration is primarily due to the over-accumulation and coarsening of primary Si and eutectic silicon, as evidenced in Figure 3d,e and Figure 4e, which disrupt the continuous α-Al matrix and act as stress concentrators under tensile loading.

Figure 7 shows the variation in Vickers hardness of the alloys as a function of the Ni-CNT content. In contrast to the trends observed in strength and ductility, hardness generally increases with increasing Ni-CNT contents, reaching a peak value of 88.75 MPa at 0.4 wt.%, corresponding to a 14.15% increase relative to the unmodified alloy (77.75 MPa). Interestingly, a slight drop in hardness is observed at 0.1 wt.% Ni-CNTs (76.75 MPa), consistent with the dominance of fine eutectic Si and a more ductile matrix structure. At higher additions (≥0.2 wt.%), the increased volume fraction and size of primary Si- and Fe/Ni-containing intermetallic phases contribute to enhanced resistance against localized plastic deformation, as these hard and brittle phases increase the alloy’s compressive stiffness. These results underscore a trade-off between ductility and hardness, determined by the relative fraction and morphology of reinforcing phases.

Fracture surface analysis via SEM (Figure 8) further supports the mechanical property evolution. At 0.1 wt.% Ni-CNTs, the fracture surface displays predominantly ductile features, including river-like patterns, shallow dimples, and uniformly distributed tearing ridges with characteristic spacings of 5–10 μm. The presence of uniformly dispersed second-phase particles in the fracture zone and a fragmented morphology of eutectic Si indicate a crack propagation path through a refined and toughened matrix, consistent with the enhanced tensile ductility observed. In contrast, at 0.2 wt.% Ni-CNTs, the fracture surface morphology shifts markedly to a brittle mode, with large, flat cleavage planes, extensive cleavage steps, and minimal evidence of plastic deformation. The brittle nature is attributed to the formation of coarse, plate-like eutectic silicon and primary Si clusters that act as crack initiation sites and facilitate intergranular cleavage. These microstructural features correspond well with the observed decreased elongation and strength shown in Figure 6, illustrating a clear microstructure–property–fracture correlation.

### 3.3. Electrical Conductivity

**Figure 9:**
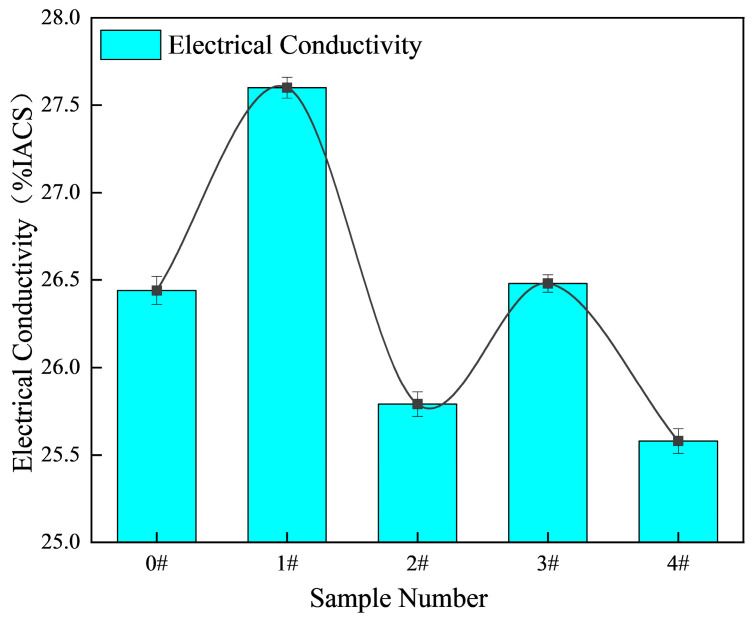
Electrical conductivity of hypereutectic Al-Si alloys as a function of Ni-CNT content.

Figure 9 presents the variation in electrical conductivity of the hypereutectic Al-Si alloys as a function of Ni-CNT content (0–0.4 wt.%). The measured conductivity values are expressed as a percentage of the International Annealed Copper Standard (% IACS), reflecting the material’s ability to conduct electric currents relative to pure copper. The unmodified alloy (0 wt.% Ni-CNTs) exhibits an electrical conductivity of 26.44% IACS, which is consistent with typical values for hypereutectic Al-Si alloys containing a substantial volume fraction of Si and intermetallic compounds that hinder electron transport. Upon the addition of 0.1 wt.% Ni-CNTs, the conductivity increases to 27.60% IACS, marking a notable enhancement of approximately 4.39%. This improvement is attributed to the refinement of the eutectic structure and α-Al matrix, as shown in Figure 3b, which enhances the continuity of the metallic matrix and reduces electron scattering at heterogeneous interfaces. The effective dispersion of Ni-CNTs promotes a more homogeneous solidification front, minimizes solute segregation, and facilitates better connectivity within the Al matrix, which is critical for maintaining high electrical conductivity.

However, with further increases in Ni-CNT content, the conductivity begins to decline. At 0.2 wt.%, the conductivity drops to 25.79% IACS, which is already 2.45% lower than the peak value and 0.65% below the base alloy. This decline continues with increasing Ni-CNT additions, reaching 25.58% IACS at 0.4 wt.%, which corresponds to a total decrease of 3.25% compared to the original alloy. This degradation is closely associated with the deterioration in microstructural uniformity at higher reinforcement contents. As observed in Figure 3d,e and Figure 4, excessive Ni-CNT addition leads to pronounced coarsening of primary Si particles and the agglomeration of brittle intermetallic phases such as Al_9_FeNi and Al_3_Ni, which are known to impede electron mobility due to their poor electrical conductivity and the increased interface density that they introduce. Furthermore, the higher volume fraction of coarse eutectic and primary silicon phases at ≥0.2 wt.% Ni-CNTs increases the electron scattering sites, thereby disrupting current pathways in the α-Al matrix. The interface between the ceramic-like Ni-CNTs and the metallic matrix may also act as an additional barrier to electron flow when clustering or poor interfacial bonding occurs. These microstructural features collectively offset the initial gains achieved at low Ni-CNT contents. It is also noteworthy that although carbon nanotubes intrinsically possess high electrical conductivity, their beneficial effect is only realized when they are well dispersed and effectively bonded within the metallic matrix. At higher concentrations, the tendency for Ni-CNTs to cluster and promote intermetallic phase formation outweighs their intrinsic conductivity, resulting in the observed decline in bulk conductivity.

## 4. Discussion

### 4.1. Phase Transformation and Nucleation Mechanism Induced by Ni-CNTs

**Figure 10:**
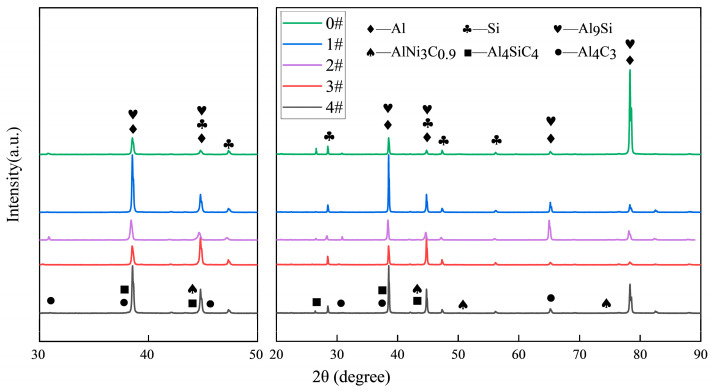
XRD patterns of hypereutectic Al-Si alloys with different Ni-CNT additions.

The addition of Ni-coated carbon nanotubes (Ni-CNTs) significantly modifies the microstructural evolution of hypereutectic Al-Si alloys. X-ray diffraction analysis (Figure 10) reveals the presence of new diffraction peaks corresponding to interfacial reaction products such as AlNi_3_C_0.9_, Al_4_C_3_, and Al_4_SiC_4_ with increasing Ni-CNT contents, suggesting chemical interactions between the Ni-CNT reinforcement and the Al-Si matrix during solidification. These phases replace part of the Al and Al_9_Si peaks, indicating a partial transformation of matrix phases under Ni-CNT-induced local compositional and energetic perturbations [34]. There is also a small amount of α-Al_8_Fe_2_Si, β-Al_5_FeSi iron phases, and copper phase compounds (Figure 5) in the XRD data. Due to the weak signal and overlapping nature of these phases, this section only discusses the effect of Ni-CNTs on the Al-Si alloy’s transformation and the induction mechanism.

The formation of these interfacial compounds is thermodynamically favored due to the highly reactive Al-C and Al-Ni interfaces in the melt. The Ni layer improves CNTs’ wettability, suppresses direct Al-C interfacial energy mismatch, and facilitates the nucleation of intermediate ternary phases such as AlNi_3_C_0_._9_. These reactions contribute not only to interfacial bonding but also to nucleation site generation. From a crystallographic perspective, the (002) graphite plane of CNTs (~0.34 nm) exhibits partial lattice matching with the {111} plane of face-centered cubic Al (~0.23 nm), enabling semi-coherent heterogeneous nucleation. This promotes refined α-Al dendrites and favors the dispersion of eutectic Si.

**Figure 11:**
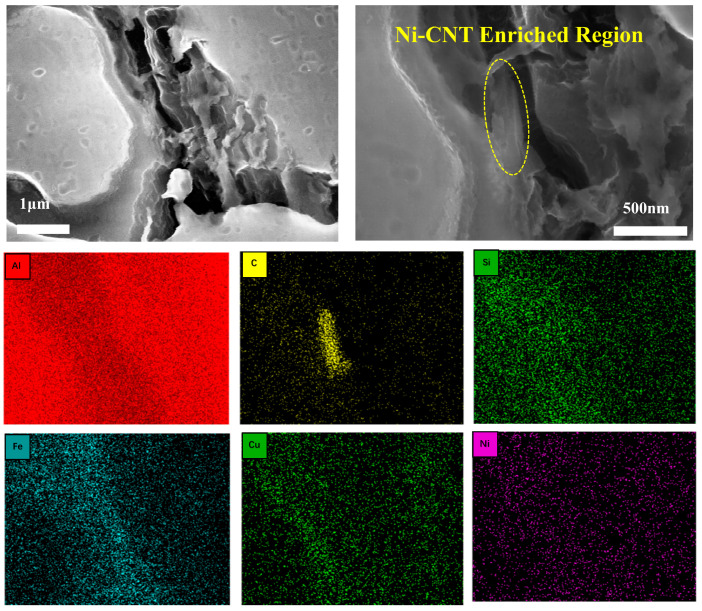
SEM image showing the microstructural distribution of Ni-CNTs in the 0.4 wt.% Ni-CNT-modified Al-Si alloy.

Scanning electron microscopy (Figure 11) shows that Ni-CNTs are embedded at grain boundaries and occasionally at eutectic Si-rich zones, forming nanolayered structures with distinct contrast. The interface transition zone suggests effective bonding but also reveals structural rigidity mismatches. In areas with excessive CNT clustering (at 0.4 wt.%), large cleavage steps (Figure 8) and coarse lamellar Si dominate, indicating that interfacial phases can serve as crack initiation sites if not properly dispersed.

**Figure 12:**
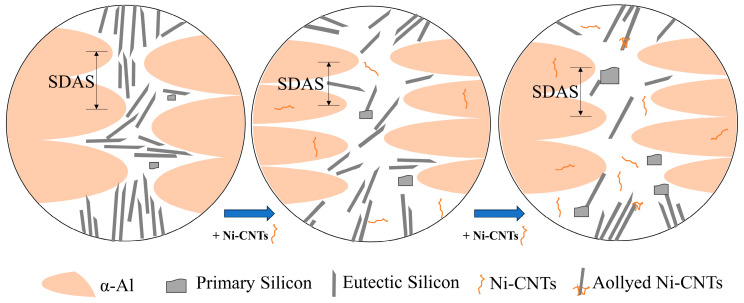
Schematic illustration of the microstructural refinement mechanism induced by Ni-CNT addition in hypereutectic Al-Si alloys.

Metallographically, Ni-CNTs act as dual-phase nucleation agents for α-Al and primary Si. Figure 12 shows that at moderate contents (0.1–0.2 wt.%), Ni-CNTs facilitate α-Al grain refinement and spheroidization of eutectic Si, which collectively improve the mechanical integrity. However, at higher contents, these Ni-CNTs act as sites for Si accumulation, leading to coarse blocky or fibrous primary Si and the loss of microstructural uniformity. These changes imply a solute-driven constitutional undercooling effect that is exacerbated by an uneven Ni-CNT distribution and local supersaturation of Si.

Overall, the presence of Ni-CNTs alters the solidification dynamics by reducing interfacial energy, increasing nucleation density, and modifying the thermodynamic equilibrium pathways between solid and liquid phases. However, excessive addition promotes interfacial phase overgrowth, localized strain accumulation, and embrittlement along eutectic zones.

### 4.2. Strength–Conductivity Coupling Mechanism Under Ni-CNT Regulation

The incorporation of Ni-CNTs significantly influences both the mechanical strength and electrical conductivity of the Al-Si alloys, governed by an interplay of microstructure refinement, dislocation dynamics, and interfacial electron transport. The results indicate that an optimal Ni-CNT content (0.1 wt.%) leads to peak performance in both tensile strength and electrical conductivity (Figure 7 and Figure 9), while further addition causes degradation due to microstructural inhomogeneity and interfacial scattering.

From a strengthening perspective, grain refinement caused by enhanced heterogeneous nucleation increases the grain boundary density, contributing to Hall–Petch-type strengthening. In addition, Orowan strengthening is activated as dislocations are forced to bypass nanoscale Ni-CNTs and intermetallic phases that are dispersed throughout the matrix. The stress required for bypassing, which scales inversely with interparticle spacing, is maximized at moderate Ni-CNT contents, where dispersion is most uniform. Furthermore, the thermal expansion mismatch between Ni-CNTs and the aluminum matrix creates residual thermal stress during cooling [35]. This mismatch is expressed as follows:

(1) $$\varepsilon =(\alpha_{CNT}-\alpha_{Al})\cdot \Delta T$$

where *α_Al_* and *α_CNT_* represent the thermal expansion coefficients between the aluminum matrix and the Ni-CNTs, and Δ*T* denotes the temperature change during solidification and cooling. Ni-CNTs generate dislocation networks and plastic deformation zones around CNTs. These dislocations accumulate at the Ni-CNT/matrix interface and contribute to dislocation strengthening, synergistically reinforcing the load-bearing capability of the composite. SEM observations (Figure 8) of cleavage surfaces at high Ni-CNT contents indicate that excessive local stress and brittle phase accumulation compromise these benefits by initiating crack propagation at grain boundaries and phase boundaries.

(2) $$\Delta \sigma_{Orowan}=\frac{0.13\cdot G\cdot b}{\lambda }\cdot \ln\left(\frac{r}{b}\right)$$

where *G* is the shear modulus of the aluminum matrix (~26 GPa), *b* is the Burgers vector (~0.286 nm for Al), *λ* is the average interparticle spacing between reinforcement phases (such as Ni-CNTs or in situ intermetallic), and *r* is the mean radius of the dispersed strengthening particles. The logarithmic term accounts for the curvature effect in dislocation bypass.

This equation indicates that the Orowan stress increases with decreasing *λ* and particle size, underscoring the importance of fine and uniform dispersion. At low Ni-CNT contents (0.1 wt.%), the interparticle spacing is minimized, maximizing dislocation bowing stress and thereby contributing significantly to the overall composite strength.

In parallel, the electrical conductivity is improved by Ni-CNT addition at low levels due to matrix refinement and the high intrinsic conductivity of Ni-CNTs. According to Matthiessen’s Rule,

(3) $$\rho_{\;total}=\rho_{matrix}+\rho_{CNT}+\rho_{interface}+\rho_{defects}$$

Here, *ρ*_matrix_ represents the intrinsic resistivity of the aluminum matrix, *ρ*_defects_ accounts for scattering from lattice imperfections such as dislocations and grain boundaries, *ρ*_interface_ is attributed to interfacial regions involving second phases (e.g., Al_4_C_3_, AlNi_3_) and particle boundaries, and *ρ*_CNT_ reflects the influence of the carbon nanotube network.

At 0.1 wt.%, the conductivity increases due to reduced *ρ*_matrix_ and *ρ*_interface_, as Ni-CNTs bridge inter-dendritic regions and reduce the resistivity associated with eutectic Si. The Ni coating improves compatibility and bonding with the Al matrix, reducing electron scattering at the interface. However, as the Ni-CNT content increases beyond 0.2 wt.%, the conductivity declines due to increased *ρ*_interface_, *ρ*_defects_ arising from Ni-CNT agglomeration, coarse primary Si, and ceramic-phase formation. These factors disrupt the continuity of the electron transport network and enhance phonon and defect scattering.

**Figure 13:**
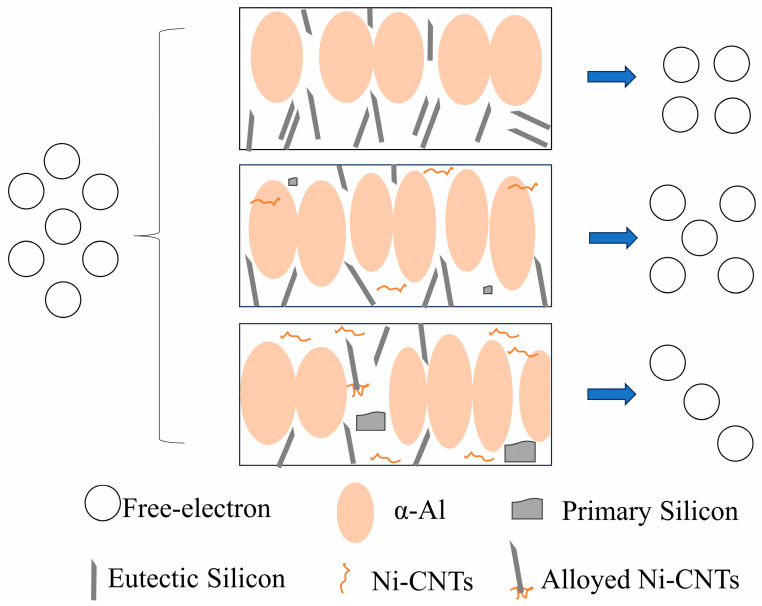
Schematic of the electro-mechanical enhancement mechanism associated with Ni-CNT incorporation in hypereutectic Al-Si alloys.

Hence, both the mechanical and conductive responses exhibit non-linear trends with the Ni-CNT content, defined by an optimum reinforcement threshold (Figure 13). Below this threshold, the coupling of grain refinement, dislocation strengthening, and interface continuity enhances performance; beyond it, phase coarsening and interfacial degradation dominate. This establishes a composition–processing–property relationship governed by nanoscale interfacial engineering.

### 4.3. Thermodynamic Effects and Solidification Behavior Modified by Ni-CNTs

**Figure 14:**
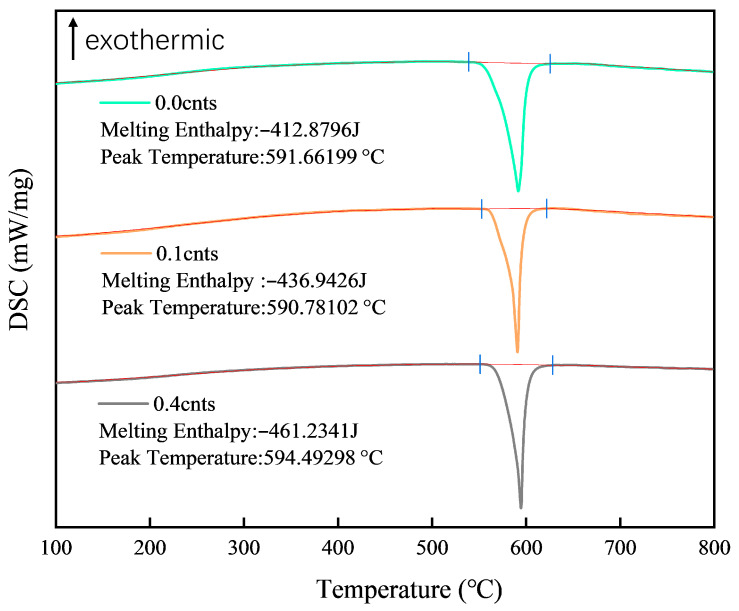
DSC curves of hypereutectic Al-Si alloys before and after Ni-CNT addition.

The thermal response of the nickel-alloyed hypereutectic Al-Si alloys, as revealed by the DSC results in Figure 14, exhibits significant dependence on the amount of Ni-coated carbon nanotubes (Ni-CNTs) that are added. The melting enthalpy increases consistently with the Ni-CNT content, rising from 412.88 J/g (0 wt.%) to 461.23 J/g (0.4 wt.%), reflecting enhanced thermal stability and increased heat absorption capacity due to the formation of high-enthalpy, thermodynamically stable interfacial phases such as AlNi_3_C_0.9_, Al_4_SiC_4_, and Al_4_C_3_. These compounds not only consume latent heat during their formation and dissolution but also limit grain boundary mobility, contributing to solid–liquid interface stabilization.

Interestingly, the melting point exhibits non-monotonic behavior, decreasing slightly from 591.66 °C to 590.79 °C at 0.1 wt.% Ni-CNTs and then increasing to 594.49 °C at 0.4 wt.%. The initial drop can be attributed to grain refinement and enhanced nucleation undercooling, induced by well-dispersed Ni-CNTs, which reduce the interfacial energy and promote earlier solidification. However, as the Ni-CNT content increases, the accumulation of high-temperature, refractory interfacial products reverse this trend, elevating the melting point due to an altered solidification pathway that is dominated by these phases.

This thermodynamic behavior is also influenced by residual stress fields arising from thermal mismatch [36]. The substantial difference between the thermal expansion coefficients of Ni-CNTs (~0.6 × 10^−6^ K^−1^) and the aluminum matrix (~23.6 × 10^−6^ K^−1^) causes interfacial strain during cooling. The resulting strain mismatch (ε) can be expressed as shown in Formula (1). This mismatch induces plastic deformation in the matrix and leads to the accumulation of dislocations, quantified by the following:

(4) $$\rho_{d}=\alpha \cdot \frac{G_{m}(\alpha_{p}-\alpha_{M})\Delta T}{b_{m}t}$$

where *ρ_d_* is the dislocation density, *α* is the dislocation strengthening coefficient (taken as 1.4 for composites), *G_m_* is the shear modulus of aluminum, *b_m_* is the Burgers vector of the matrix, and *t* is the effective Ni-CNT thickness.

The generated dislocation network strengthens the matrix via a Taylor-type relationship [37]:

(5) $$\Delta \sigma =M\cdot \alpha \cdot G_{m}\cdot b_{m}\cdot \sqrt{\rho_{d}}$$

where *M* is the Taylor factor. This dislocation density, especially when localized at the Ni-CNT/matrix interface, synergizes with Orowan strengthening mechanisms by hindering dislocation motion, further enhancing yield strength and thermal resistance.

Additionally, the CNTs’ rigidity and their distribution in the eutectic Si-rich regions alter the local thermal gradient during solidification, promoting anisotropic phase growth and shifting the equilibrium path. While moderate Ni-CNT addition facilitates controlled nucleation and refines the microstructure, excessive incorporation leads to interfacial saturation, fluidity loss, and higher cracking tendency during casting due to constrained shrinkage accommodation.

The addition of Ni-CNTs introduces a complex and highly interrelated thermodynamic effect: it not only modifies the heat absorption and solidification front morphology but also acts as a source of internal stress and dislocation strengthening. The balance between these competing effects must be carefully optimized to tailor the solidification behavior and structural integrity of Ni-alloyed hypereutectic Al-Si alloys.

## 5. Conclusions

This study systematically investigated the role of interface-engineered Ni-CNTs in tailoring the microstructure and enhancing the multifunctional performance of hypereutectic Al-20Si alloys. Several conclusions can be drawn:

(1) Ni-CNTs act as effective heterogeneous nucleation sites for both α-Al and primary Si phases. Their presence promotes the refinement of α-Al dendrites and transformation of eutectic Si from coarse lamellae to a more spheroidized and uniformly distributed morphology. XRD and SEM-EDS analyses confirmed the formation of interfacial reaction phases including AlNi_3_C_0.9_, Al_4_C_3_, and Al_4_SiC_4_, which alter the solidification pathway and stabilize the microstructure thermodynamically.
(2) The mechanical enhancements at a low Ni-CNT content (0.1 wt.%) are attributed to multiple synergistic mechanisms, including grain refinement, Orowan looping, load transfer, and the generation of dislocations induced by thermal mismatch between the matrix and the Ni-CNTs. These effects lead to an optimized microstructure with higher tensile strength (170.13 MPa) and ductility (5.64%). However, excessive Ni-CNT addition results in agglomeration, phase coarsening, and brittle interfacial structures that deteriorate mechanical performance.
(3) The electrical conductivity exhibits a peak at 0.1 wt.% Ni-CNTs (27.60% IACS), which is governed by improved α-Al continuity, refinement of eutectic Si, and the conductive nature of dispersed Ni-CNTs. Beyond this threshold, interface scattering, defect accumulation, and secondary phase agglomeration disrupt the conduction network, leading to a gradual decline in conductivity in accordance with Matthiessen’s Rule.
(4) DSC analysis demonstrated that Ni-CNTs increase the melting enthalpy and slightly shift the melting point via nucleation-induced undercooling and in situ phase formation. At high Ni-CNT contents, the formation of refractory phases and thermal stress accumulation result in solidification hindrance, decreased fluidity, and potential casting defects, revealing a complex interplay between thermal mismatch strengthening and phase transformation energetics.
(5) The incorporation of Ni-CNTs provides an effective strategy for realizing strength–conductivity synergy in Al-Si alloys through precise nanoscale interface engineering. By optimizing the Ni-CNT content and dispersion state, it is possible to simultaneously achieve grain refinement, interfacial reinforcement, thermal stability, and electronic continuity. These findings offer important insights for the design of high-performance, sustainable aluminum matrix composites with multifunctional capabilities, particularly for structural–functional integration in automotive and aerospace sectors.

## Figures and Tables

**Table 1:** Aluminum matrix composites’ compositions.

Sample No.	Nominal Compositions (wt.%)/Actual Compositions (wt.%)							
	Nickel-Plated CNTs	Si	Zn	Fe	Cu	Ni	Mn	Al
#1	0	20/19.94	1.30/1.21	0.80/1.32	0.70/1.02	0.30/0.29	0.10/0.21	76.80/76.01
#2	0.1	20/19.78	1.30/0.96	0.80/1.33	0.70/0.84	0.30/0.41	0.10/0.19	76.70/76.49
#3	0.2	20/20.06	1.30/1.12	0.80/1.42	0.70/0.83	0.30/0.44	0.10/0.13	76.60/76.00
#4	0.3	20/20.15	1.30/1.01	0.80/1.17	0.70/0.79	0.30/0.38	0.10/0.14	76.50/76.36
#5	0.4	20/20.01	1.30/0.98	0.80/1.46	0.70/1.06	0.30/0.34	0.10/0.26	76.40/75.89

Note: The first value in each column refers to the nominal (designed) composition, and the second indicates the actual value, measured by a direct-reading optical emission spectrometer (ThermoFisher/iSpark 8860, Thermo Fisher Scientific, Waltham, MA, USA). All measurements were repeated at least five times per sample. Results are accurate to the last significant digit, with a measurement uncertainty within ±0.02 wt.%.

**Table 2:** EDS analysis of each point in Figure 5 (Unit: wt.%).

Point	Al	Si	Fe	Ni	Zn	Cu	Possible Phase
A	43.71	55.70	0.08	0.04	0.47	\	Eutectic Si
B	65.99	9.70	23.00	0.77	0.54	\	α-Al8Fe2Si
C	67.30	2.04	5.99	19.70	1.37	3.60	Al9FeNi + Al3Ni
D	65.15	13.65	19.10	1.62	0.47	\	β-Al5FeSi
E	18.37	81.05	0.01	0.05	0.18	0.33	Primary Si
F	64.18	7.77	22.39	0.65	4.08	0.94	α-Al8Fe2Si

## Data Availability

The original contributions presented in this study are included in the article. Further inquiries can be directed to the corresponding authors.

## Abbreviations

The following abbreviations are used in this manuscript:

DSC	Differential Scanning Calorimetry
SEM	Scanning Electron Microscope
XRD	X-Ray Diffraction
SDAS	Secondary Dendrite Arm Space
(Ni-CNTs)	Nickel-Coated Carbon Nanotubes
